# Gut Microbiota–Mucosal Immune Interactions Underlying Intestinal Homeostasis in Weaned Piglets

**DOI:** 10.3390/ani16193075

**Published:** 2026-09-30

**Authors:** Jieyi Qin, Tiantian Li, Xiao Li, Peiyuan Han, Mingliang Jin, Lei Qiao

**Affiliations:** 1 Key Laboratory of Molecular Animal Nutrition of the Ministry of Education, College of Animal Sciences, Zhejiang University, Hangzhou 310058, China; 22417011@zju.edu.cn (J.Q.); 22417047@zju.edu.cn (X.L.); 12617027@zju.edu.cn (P.H.); 2 School of Life Science and Technology, Northwestern Polytechnical University, Xi’an 710129, China; tianli@mail.nwpu.edu.cn

**Keywords:** weaned piglets, gut microbiota, mucosal immunity, microbiota–immune interactions, intestinal homeostasis, nutritional modulation

## Abstract

The gut microbiota and mucosal immune system undergo extensive remodeling after weaning and jointly influence intestinal homeostasis, resistance to enteric pathogens, and growth performance in piglets. This developmental transition is particularly vulnerable to dietary changes, the loss of maternally derived factors, environmental stressors, and pathogen exposure, all of which can disrupt microbial ecology and mucosal immune regulation. This review examines the reciprocal interactions between the gut microbiota and mucosal immunity in weaned piglets. It describes how microbial communities and their metabolites regulate epithelial barrier integrity, innate and adaptive immune responses, B-cell differentiation, and secretory immunoglobulin A (sIgA) production, with particular emphasis on short-chain fatty acids, tryptophan-derived metabolites, and bile acids. Conversely, host-derived factors, including mucins, antimicrobial peptides, sIgA, cytokines, and inflammation-associated changes in the intestinal microenvironment, are discussed in relation to microbial colonization, spatial distribution, and functional activity. Disruption of these interactions can impair epithelial barrier function, promote excessive inflammation, weaken colonization resistance, and increase susceptibility to enteric infection and post-weaning diarrhea. Finally, this review evaluates nutritional and microbiota-targeted strategies for modulating microbiota–immune interactions and improving intestinal health in weaned piglets.

## Simple Summary

The post-weaning period is a critical stage in pig production. Abrupt changes in diet, environment, and microbial exposure can disrupt intestinal homeostasis, increasing the risk of diarrhea and impaired growth. The gut microbiota interacts closely with the intestinal immune system to maintain barrier integrity, regulate immune responses, and limit pathogen colonization. This review summarizes the reciprocal interactions between the gut microbiota and mucosal immunity in weaned piglets and examines how their disruption contributes to intestinal dysfunction. It also discusses nutritional and microbiota-targeted strategies for promoting beneficial microbial communities and improving intestinal health. A better understanding of these interactions may support the development of effective nutritional interventions for healthy growth and sustainable pig production.

## 1. Introduction

Weaning represents a major developmental and ecological transition for piglets. The duration of the suckling period varies among pig-production systems. Piglets are generally weaned at 21–28 days of age in conventional production, whereas organic systems commonly maintain suckling for at least 40 days [1]. Under commercial production conditions, separation from the sow coincides with the withdrawal of milk and maternally derived bioactive factors, the introduction of solid feed, social regrouping, and transfer to a new housing environment. These changes occur before digestive capacity, epithelial barrier function, and mucosal immunity are fully mature [2,3]. Consequently, the immediate post-weaning period is commonly characterized by reduced feed intake, altered villus–crypt architecture, impaired digestion and nutrient absorption, increased intestinal permeability, and changes in mucosal immune activity [4]. Although many of these responses are transient, inadequate adaptation can increase susceptibility to enteric infection and post-weaning diarrhea, thereby compromising growth performance and feed efficiency [5]. Intestinal homeostasis during this period therefore depends on the capacity of the gastrointestinal tract to accommodate concurrent nutritional, microbial, and immunological changes.

The gut microbiota also undergoes extensive remodeling during the transition from suckling to weaning. The replacement of milk-derived substrates with plant proteins and complex carbohydrates, together with changes in maternal contact, environmental exposure, and intestinal physiology, alters microbial composition, substrate utilization, and metabolite production [6]. Longitudinal studies indicate that diet and weaning are major determinants of this ecological succession and that nutritional exposure before weaning can influence its subsequent trajectory [7,8]. However, compositional shifts after weaning should not automatically be interpreted as dysbiosis, as much of this succession reflects physiological adaptation to a new intestinal environment. Evidence of maladaptation is better inferred from community instability, loss of beneficial microbial functions, altered metabolite production, reduced colonization resistance, or disrupted spatial organization. Importantly, microbial remodeling coincides with epithelial and immune maturation. Studies integrating microbiota profiling with intestinal morphology, host gene expression, or metabolomics have demonstrated close temporal associations among these processes, although the underlying causal relationships remain incompletely understood [9].

The intestinal microbiota and mucosal immune system therefore function as a reciprocal regulatory network rather than as independent determinants of gut health. Microbial structural components and metabolites are sensed by epithelial and immune cells, thereby influencing epithelial renewal, mucin production, antimicrobial defense, innate immune responsiveness, lymphocyte differentiation, and mucosal antibody responses [10,11]. Conversely, the host shapes the microbial habitat through the secretion of mucins, antimicrobial peptides, secretory immunoglobulin A (sIgA), and cytokines, as well as through changes in nutrient availability and physicochemical conditions at the mucosal surface [12,13,14]. Collectively, these host responses limit inappropriate microbial contact with the epithelium, promote tolerance to resident microorganisms, and preserve responsiveness to invading pathogens. Their effects are highly context-dependent. Appropriately regulated immune responses facilitate barrier repair and pathogen clearance, whereas excessive or prolonged activation can cause tissue injury and disrupt the intestinal microbial ecosystem. Disturbance of these reciprocal interactions can establish a self-reinforcing cycle in which barrier impairment increases mucosal exposure to microbial products, inflammation alters the intestinal habitat, and the resulting environmental changes further reduce microbial community stability and colonization resistance [15]. In a controlled challenge study using F18 enterotoxigenic *Escherichia coli* (ETEC), piglets weaned at 16 or 18 days of age, defined as early-weaned relative to the 20-day group, exhibited attenuated innate mucosal responses and more severe intestinal injury and diarrhea [4]. Microbiota-transfer studies further indicate that differences in microbial community composition can influence diarrhea susceptibility, supporting the biological importance of microbiota–immune interactions during weaning [16].

Previous reviews have described post-weaning microbial succession, barrier dysfunction, microbial metabolites, and nutritional interventions in considerable detail [5,10,17]. A recent review has also provided a broad discussion of host–microbiota interactions, including metabolic and neuroendocrine regulation, multi-omics approaches, and nutritional strategies [17]. The present review concentrates on a narrower aspect of this field: the reciprocal relationship between the gut microbiota and mucosal immunity. The emphasis is placed on the mechanisms linking microbial signals, epithelial responses, and immune-cell activity, rather than on parallel descriptions of microbial taxa, barrier indices, cytokine profiles, and growth performance.

**Figure 1:**
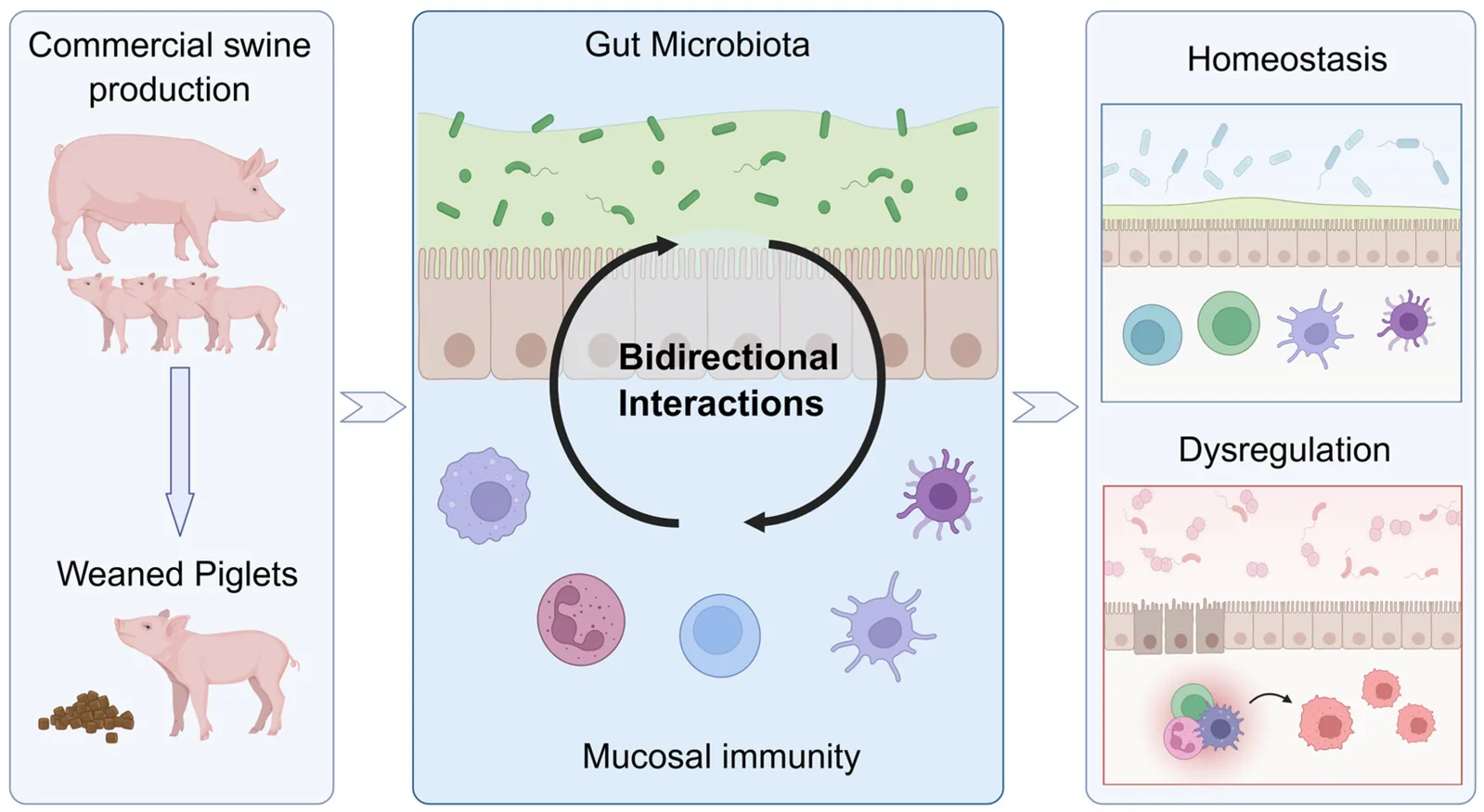
Bidirectional microbiota–mucosal immune regulation in weaned piglets. Microbial ligands and metabolites regulate epithelial barrier function and innate and adaptive immune responses. In turn, mucus, antimicrobial peptides, sIgA, and cytokine-induced changes in the intestinal habitat influence microbial persistence, spatial localization, and functional activity. Balanced feedback supports intestinal homeostasis, whereas disruption of this regulatory loop promotes barrier injury, inflammation, loss of colonization resistance, pathogen expansion, and post-weaning diarrhea.

Figure 1 summarizes the reciprocal interactions discussed in this review. Microbial ligands and metabolites are sensed by epithelial and immune cells and influence barrier integrity, innate immune activity, type 3 immune responses, regulatory T-cell differentiation, and IgA-producing B-cell responses. Conversely, mucus, antimicrobial peptides, sIgA, cytokines, and inflammation-associated changes in the intestinal environment shape microbial colonization, spatial distribution, and function. When these interactions remain coordinated, they support intestinal homeostasis; when disrupted, they can allow barrier injury, inflammation, reduced colonization resistance, and pathogen expansion to reinforce one another after weaning. The subsequent sections examine these processes and the points at which nutritional interventions may act.

## 2. Review Methodology

This narrative review synthesized evidence on reciprocal interactions among the gut microbiota, microbial metabolites, the epithelial barrier, and mucosal immunity in weaned piglets. A structured literature search was conducted in PubMed, Web of Science Core Collection, and Scopus for English-language articles published between 1 January 2000 and 21 September 2026. Google Scholar was used as a supplementary source for keyword searches and citation tracking. The final search was performed on 21 September 2026. Search terms were adapted to the syntax and field options of each database and combined using the Boolean operators AND and OR, together with truncation where applicable. A population block comprising piglet*, “weaned piglet*”, and swine was combined with seven thematic blocks addressing post-weaning microbial remodeling, epithelial barrier regulation, mucosal immune regulation, immune-mediated microbial selection, microbial metabolites, dysbiosis and enteric disease, and nutritional modulation.

After deduplication across all sources, 1986 unique records were screened by title and abstract, followed by full-text assessment of potentially eligible articles. Studies were eligible if they investigated microbial succession or function during weaning; interactions of the microbiota with the epithelial barrier or mucosal immune system; immune regulation of microbial colonization; microbial metabolites involved in mucosal regulation; post-weaning dysbiosis, barrier dysfunction, enteric infection, or diarrhea; or nutritional interventions reporting microbial, metabolic, epithelial, or immune outcomes. In vivo studies in piglets constituted the primary evidence base. Evidence from rodents, humans, and non-porcine cell systems was included when the corresponding mechanism had not been adequately resolved in pigs, and the experimental origin of such evidence was identified in the text. These non-porcine mechanistic studies were identified by tracing references and citing articles of eligible piglet studies and relevant reviews, without applying the porcine population restriction. Selection focused on studies directly examining the microbial signals, host receptors, or cellular mechanisms discussed in this review. Studies unrelated to intestinal microbial ecology or mucosal function, studies reporting growth performance without relevant intestinal outcomes, conference abstracts, dissertations, non-peer-reviewed reports, non-English publications, and articles for which the full text could not be retrieved were excluded.

Overall, 199 publications were included in the narrative synthesis. Evidence was organized into five themes: post-weaning remodeling, microbiota-to-host regulation, host-to-microbiota regulation, disruption of microbiota–immune interactions, and nutritional modulation. Given the heterogeneity in experimental design, sampling procedures, analytical methods, and reported outcomes, evidence was integrated through qualitative thematic synthesis rather than meta-analysis. As the review used only previously published evidence, ethical approval was not required. To provide a concise overview, Table 1 summarizes the guiding question and key message of each section, from the rationale and methodology of the review to post-weaning intestinal remodeling, reciprocal microbiota–mucosal immune regulation, dysregulation, nutritional modulation, and future research priorities.

## 3. Post-Weaning Remodeling of the Gut Microbiota and Mucosal Immune System

Under commercial production conditions, piglets are generally weaned before the gut microbiota, epithelial barrier, and mucosal immune system have fully matured [18]. The abrupt withdrawal of sow’s milk removes a major source of readily digestible nutrients and maternally derived bioactive and immune factors [3]. Meanwhile, separation from the sow, social regrouping, and transfer to a new housing environment introduce additional physiological and environmental stressors. Microbial succession, epithelial adaptation, and mucosal immune maturation therefore occur concurrently during the early post-weaning period, and the interactions among these processes are central to the establishment of intestinal homeostasis [9,10].

### 3.1. Remodeling of the Gut Microbiota After Weaning

During suckling, the gut microbiota develops within a milk-based nutritional environment rich in lactose, lipids, and highly digestible proteins and is also exposed to milk oligosaccharides and glycoprotein-associated glycans [7,19]. Access to creep feed may initiate microbial adaptation to solid substrates before weaning, whereas the complete withdrawal of sow’s milk shifts substrate availability toward starch, non-starch polysaccharides, and plant proteins [7,20]. Longitudinal studies identify this dietary transition as a major inflection point in microbial development. Metagenomes from suckling piglets are enriched in gene families associated with lactose and galactose utilization, whereas post-weaning communities show greater representation of functions related to plant-derived carbohydrate degradation and amino acid metabolism [7,20]. The most pronounced compositional reorganization is often observed within the first week after weaning, followed by continued maturation throughout the nursery period [21,22,23]. Longitudinal studies based on fecal samples have generally reported a decline in suckling-associated Bacteroides and an expansion of *Prevotella* after the introduction of solid feed, with *Prevotella copri* becoming prominent during the nursery period [7,21,22]. Increases in the lactate-utilizing species *Megasphaera elsdenii* and the butyrate-producing species *Butyricicoccus porcorum* and *Faecalibacterium prausnitzii* have also been detected in fecal samples within the first week after weaning [23]. Together with metagenomic evidence, these taxonomic shifts are consistent with an increasing microbial capacity for plant-polysaccharide degradation, lactate utilization, and short-chain fatty acid production.

Microbial remodeling varies considerably among intestinal regions and between luminal and mucosa-associated habitats [24]. Faster transit, exposure to bile acids, and host nutrient absorption generally limit microbial density and fermentative activity in the small intestine relative to the cecum and colon, where slower transit and strongly anaerobic conditions support dense fermentative communities. Luminal and mucosa-associated microbiota also occupy distinct ecological niches [25]. Luminal communities are strongly influenced by digesta composition and passage rate, whereas mucosa-associated communities develop under the selective pressures imposed by mucin glycans, epithelial oxygen gradients, antimicrobial peptides, and sIgA [24]. A source-tracking study of mucosa-associated communities estimated that sow-milk microorganisms contributed substantially to early colonization of the small intestine, whereas the contribution of maternal fecal microorganisms to the large-intestinal microbiota increased with age [26]. Fecal microbial profiles mainly reflect distal luminal communities and are not equivalent to profiles obtained from the ileal lumen or mucosal surface. Changes detected in feces may therefore not mirror those occurring in mucosa-associated communities, which interact more directly with the host [24,26]. This spatial organization exposes different intestinal regions to distinct combinations and concentrations of microbial antigens and metabolites [24].

Post-weaning succession is also accompanied by functional maturation of the microbial ecosystem. Enrichment of carbohydrate-active enzyme genes involved in the degradation of starch and plant cell-wall polysaccharides indicates an increased functional capacity to utilize complex dietary carbohydrates. Concurrent changes in genes associated with amino acid metabolism reflect the altered composition and intestinal availability of protein substrates after weaning [20,23]. These changes can shift the balance between saccharolytic and proteolytic fermentation and modify the intestinal concentrations of short-chain fatty acids (SCFAs), lactate, succinate, branched-chain fatty acids, ammonia, and other nitrogen-containing metabolites [27]. Microbial maturation is generally characterized by progression toward an age-associated community configuration and greater temporal stability. Functional redundancy may help preserve selected metabolic outputs despite taxonomic turnover, whereas ecological resistance and resilience may support the maintenance and recovery of community function during dietary, environmental, or infectious disturbances [28].

### 3.2. Maturation of the Intestinal Barrier and Mucosal Immune System

The intestinal mucosal barrier comprises a continuously renewed epithelial monolayer sealed by intercellular junctional complexes, an overlying mucus layer secreted by goblet cells, epithelial antimicrobial products, and underlying immune cells [29,30]. Around weaning, crypt cell proliferation, epithelial differentiation and migration, and cell shedding at the villus tip undergo rapid adjustment [31]. Piglet studies have documented a transient reduction in epithelial proliferation during the first few days after weaning and increased epithelial shedding in the jejunum, accompanied by alterations in villus–crypt architecture, tight junction proteins, and intestinal permeability [31,32,33]. Goblet cell abundance and differentiation, mucin production, and antimicrobial peptide expression also remain dynamic and vary among intestinal regions in response to age, diet, and microbial exposure [31,34]. The early post-weaning barrier phenotype therefore reflects changes in epithelial turnover and secretory defense as well as junctional organization.

Mucosal immune populations develop concurrently. Macrophages and dendritic cells (DCs) participate in microbial clearance, antigen sampling and presentation, and local immune regulation, whereas neutrophils provide rapidly recruited antimicrobial defense [35,36]. Intestinal lymphocyte populations also continue to change around weaning. Porcine intestinal group 3 innate lymphoid cell (ILC3) populations develop progressively from birth to 21 days of age, indicating that this compartment remains developmentally dynamic near conventional weaning age [37]. Intraepithelial γδ and αβ T-cell populations change in abundance and phenotype between 4 and 8 weeks of age and exhibit distinct distributions in the small and large intestine [38]. Single-cell and transcriptomic analyses have further identified age- and weaning-associated changes in epithelial, myeloid, B-cell, and T-cell states [34,39]. These findings indicate that the composition and activation state of the mucosal immune system remain dynamic as the intestine encounters increasing dietary and microbial antigen exposure.

Adaptive mucosal immunity is also undergoing maturation. Peyer’s patches and mucosal DC networks provide sites for antigen sampling, presentation, and lymphocyte activation, while T helper 17 (Th17) and regulatory T-cell populations contribute to the balance between mucosal defense and immune tolerance. Activated B cells differentiate into plasma cells that produce IgA, which is transported across the epithelium into the intestinal lumen as sIgA [39,40,41]. Weaning abruptly terminates the continuous supply of sow-milk-derived sIgA and other maternal immune factors. Ileal sIgA concentrations can decline markedly after milk withdrawal, even as endogenous intestinal IgA-producing cells increase during the subsequent post-weaning period [40,41]. This temporal mismatch may create a transient period of reduced antibody-mediated exclusion at the mucosal surface. Controlled immune activation during this period supports adaptation to newly encountered antigens and may facilitate epithelial repair, whereas excessive or persistent activation can promote inflammation and barrier injury.

### 3.3. Factors Driving Post-Weaning Intestinal Remodeling

Post-weaning intestinal remodeling is driven by several concurrent changes. The replacement of milk with solid feed alters nutrient composition, physical form, digestibility, and feeding pattern, while voluntary feed intake commonly declines during the first few days after weaning. The resulting reduction in nutrient supply decreases the availability of dietary substrates to both the epithelium and microbiota and contributes to impaired barrier function. In one controlled study, low energy intake had a greater influence on early small-intestinal integrity than variation in the dietary lactose-to-protein ratio [42]. Milk withdrawal also terminates the continuous luminal supply of maternal immunoglobulins, lactoferrin, growth factors, cytokines, and milk oligosaccharides [3]. These nutritional changes coincide with separation from the sow, relocation, mixing with unfamiliar piglets, and adaptation to a new housing environment [4]. Conversely, pre-weaning access to fibrous solid feed can accelerate microbial maturation, alter mucosal development, and modify the initial response to weaning [8,43].

The rearing environment shapes the microorganisms and antigens encountered during this developmentally sensitive period. Hygiene, housing conditions, and farm-specific microbial exposure can influence intestinal microbial communities and basal mucosal immune activity [44]. Relocation and social mixing increase exposure to unfamiliar microorganisms, while enteric infection can exacerbate inflammation, epithelial injury, and clinical disease [4]. Antibiotic exposure imposes additional selective pressure on intestinal bacterial communities, phage-associated functions, and the resistome, with effects that depend on the antimicrobial compound, treatment timing, and intestinal location [45]. Collectively, these exposures can alter microbial community stability, colonization resistance, and mucosal inflammatory tone during post-weaning adaptation.

The relative contributions of these drivers vary across production systems and experimental settings. Weaning age, basal diet, feed intake, genetic background, sanitary status, antimicrobial exposure, and pre-weaning management can all influence the observed trajectory. Surveys across commercial farms have identified a broadly shared pattern of post-weaning microbiota development together with substantial farm-associated variation [46]. Reciprocal cross-fostering experiments similarly indicate that age, solid-feed introduction, and weaning can exert stronger effects on early bacterial succession than breed or nursing mother under the conditions examined [47]. Sampling location introduces further variation because feces, intestinal digesta, and mucosa-associated communities represent distinct microbial habitats [24]. Consequently, post-weaning intestinal remodeling cannot be attributed to weaning alone but reflects the combined effects of host maturation, dietary transition, withdrawal of maternal inputs, environmental exposure, and husbandry practices.

Together, these concurrent changes provide the developmental setting for the two directions of regulation examined below: microbial signals acting on the developing mucosa and mucosal responses feeding back on microbial persistence, localization, and function.

## 4. Microbial Regulation of Mucosal Immunity in Weaned Piglets

**Figure 2:**
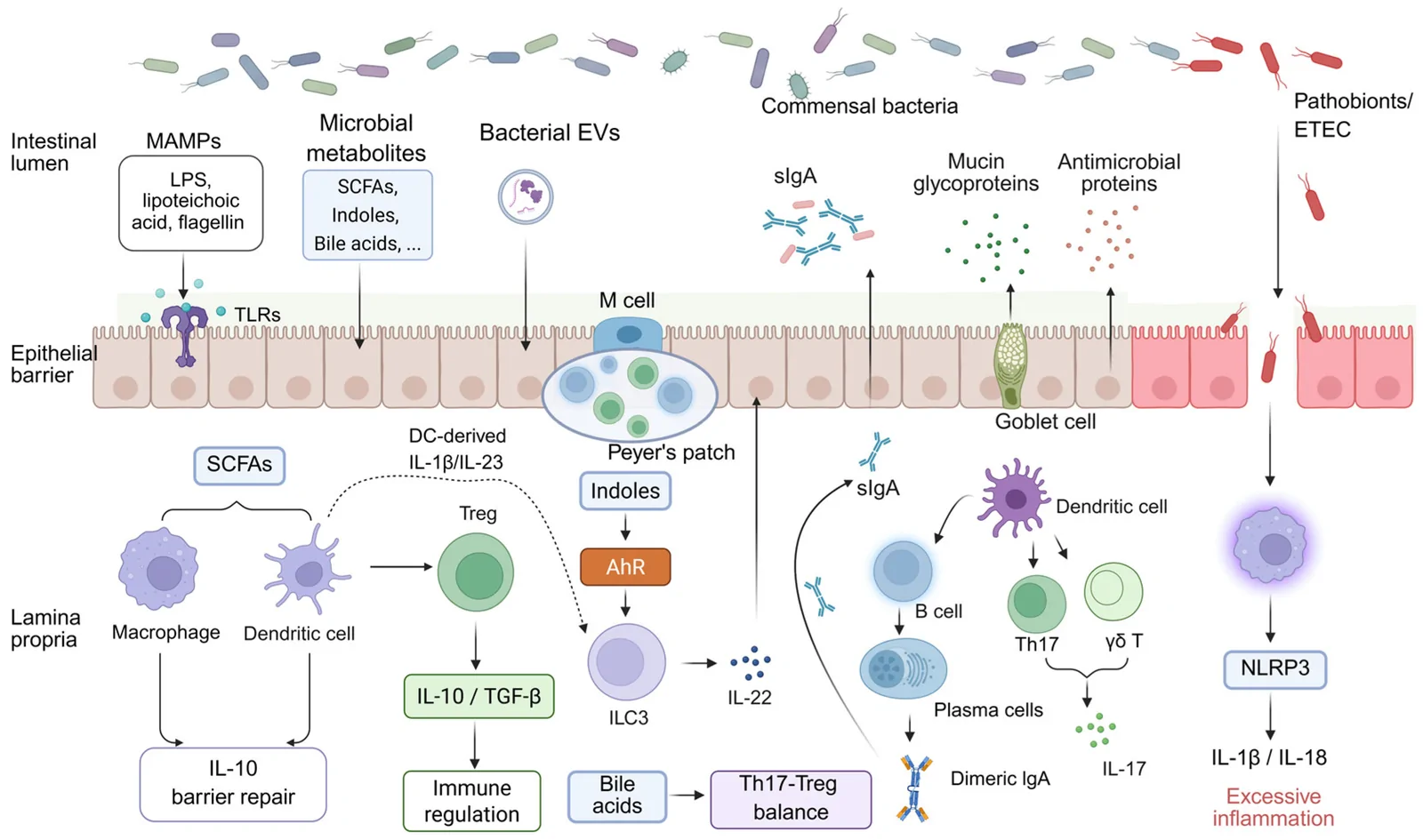
Microbial regulation of intestinal mucosal immunity in weaned piglets. Microbial structural components, extracellular vesicles, and metabolites interact with epithelial and immune cells after weaning. Short-chain fatty acids, tryptophan-derived indoles, and secondary bile acids regulate macrophage, dendritic-cell, type 3, Treg, and IgA responses, thereby supporting barrier repair, immune balance, and microbial containment. In contrast, ETEC and pathobionts can activate NLRP3-associated inflammatory responses and aggravate mucosal injury. Evidence shown in this figure comes from both porcine studies and mechanistic work in mice or human cells; the experimental model is specified in the text. Abbreviations: AhR, aryl hydrocarbon receptor; DC, dendritic cell; ETEC, enterotoxigenic *Escherichia coli*; EVs, extracellular vesicles; IgA, immunoglobulin A; IL-1β, interleukin-1 beta; IL-10, interleukin-10; IL-17, interleukin-17; IL-18, interleukin-18; IL-22, interleukin-22; IL-23, interleukin-23; ILC3, type 3 innate lymphoid cell; γδ T, gamma delta T cell; LPS, lipopolysaccharide; M cell, microfold cell; MAMPs, microbe-associated molecular patterns; NLRP3, NOD-like receptor family pyrin domain-containing 3; SCFAs, short-chain fatty acids; sIgA, secretory immunoglobulin A; TGF-β, transforming growth factor beta; Th17, T helper 17 cell; TLRs, Toll-like receptors; Treg, regulatory T cell.

Post-weaning shifts in microbial composition acquire functional significance at the mucosal interface, where epithelial and immune cells encounter microbial cell-surface components, secreted molecules, and metabolites [21,48]. The resulting response depends on the identity, intensity, duration, and spatial context of these signals [49]. At an intact epithelial surface, low-level sensing of commensal-derived signals can support epithelial renewal, barrier maintenance, and immune tolerance. In contrast, greater or sustained exposure, particularly within basolateral compartments following barrier disruption, is more likely to elicit inflammatory signaling [50]. In weaned piglets, microbial sensing occurs alongside ongoing barrier maturation and immune differentiation, thereby shaping mucosal adaptation to the altered luminal environment. As summarized in Figure 2, the balance between compartmentalized microbial sensing and excessive mucosal exposure influences whether microbial signals predominantly support barrier repair, immune regulation, and IgA-mediated containment or contribute to sustained mucosal inflammation. Direct mechanistic evidence in weaned piglets remains limited. In the following sections, evidence from piglets and porcine experimental systems is identified separately from findings in mice, human cells, or other non-porcine models. The latter are used to indicate possible mechanisms and not as direct evidence in pigs.

### 4.1. Microbial Sensing at the Intestinal Mucosal Interface

Microbe-associated molecular patterns (MAMPs), often termed pathogen-associated molecular patterns (PAMPs) in the context of infection, include lipopolysaccharide (LPS), bacterial lipoproteins, lipoteichoic acid, flagellin, peptidoglycan fragments, and microbial nucleic acids. Cell-surface Toll-like receptors (TLRs) recognize extracellular microbial components, whereas endosomal TLRs primarily detect internalized microbial nucleic acids. Nucleotide-binding oligomerization domain-containing proteins 1 and 2 (NOD1/2) function as cytosolic sensors of specific peptidoglycan derivatives. Most TLRs signal through myeloid differentiation primary response 88 (MyD88), whereas TLR3 and TLR4 can engage the TIR-domain-containing adaptor-inducing interferon-β (TRIF)-dependent pathway. NOD1/2 signal through receptor-interacting serine/threonine-protein kinase 2 (RIPK2) [51,52]. These pathways activate nuclear factor kappa B (NF-κB), mitogen-activated protein kinases (MAPKs), and, for selected TLRs, interferon regulatory factors, thereby regulating cytokines, chemokines, antimicrobial peptides, and epithelial survival and repair. Inflammasomes provide an additional layer of cytosolic surveillance. Following transcriptional priming, infection- or damage-associated cellular disturbances can activate the NLR family pyrin domain containing 3 (NLRP3) inflammasome, leading to caspase-1-dependent maturation of interleukin (IL)-1β and IL-18 [50]. These receptors and signaling relationships have been defined largely in non-porcine experimental systems. In newly weaned piglets, changes in bacterial communities have been reported alongside altered intestinal expression of TLR- and NOD-related genes [53]. Colonic transcriptomic responses are detectable within 1 day of weaning and include enrichment of TLR, NOD-like receptor, Janus kinase–signal transducer and activator of transcription (JAK–STAT), and interferon-response pathways, together with genes involved in antibacterial defense [40].

Microbial recognition is not intrinsically pro-inflammatory. In mice, basal recognition of resident microbiota through TLR–MyD88 signaling protects against chemically induced epithelial injury, while NLRP3-dependent IL-18 can support epithelial integrity during experimental colitis [54,55]. Conversely, after mucosal injury, selected resident bacteria can induce NLRP3-dependent IL-1β production by recruited monocytes and thereby exacerbate intestinal inflammation [56]. The outcome therefore depends on the microorganism and its ligands or virulence factors, as well as the location, intensity, and duration of exposure. An intact barrier largely confines microbial material to the apical surface, whereas epithelial adhesion, invasion, or translocation exposes receptors in epithelial, lamina propria, and intracellular compartments. Signal intensity is further influenced by microbial load, epithelial permeability, and the developmental and activation states of responding cells. In piglets, early weaning increases intestinal permeability and pro-inflammatory cytokine expression and activates p38 mitogen-activated protein kinase (p38), c-Jun N-terminal kinase (JNK), and extracellular signal-regulated kinases 1/2 (ERK1/2) signaling [33]. Loss of epithelial containment therefore increases the amount of microbial material encountered by the mucosa and alters the cellular compartments in which it is sensed.

### 4.2. Microbial Regulation of Epithelial and Mucus Barriers

Epithelial renewal is a central component of the intestinal response to microbial signals. Rates of proliferation, lineage differentiation, migration, apoptosis, and cell shedding change rapidly after weaning and vary along the crypt–villus axis [31]. Microbial metabolites may contribute to this remodeling. During the first 3 weeks after weaning, temporal changes in jejunal butyrate concentrations and SCFA-receptor expression were associated with concurrent changes in epithelial proliferation, apoptosis, and inflammatory gene expression [57]. Stronger intervention evidence has been obtained for indole-3-aldehyde (IAld), a microbiota-derived tryptophan metabolite. Dietary IAld supplementation increased intestinal stem-cell markers, epithelial proliferation and differentiation, villus area, the number of Mucin 2 (MUC2)-positive cells, and occludin abundance in weaned piglets. IAld also enhanced the growth of porcine small-intestinal organoids [58]. These findings support an epithelial response to IAld that encompasses tissue renewal, secretory-cell differentiation, and junctional protection.

Tight junctions regulate the paracellular passage of luminal microbial products and therefore influence their access to basolateral receptors and underlying immune cells. The tight-junction complex, comprising claudins, occludin, and zonula occludens scaffold proteins, is functionally coupled to the perijunctional actomyosin ring. Inflammatory cytokines can disrupt this organization, in part through activation of myosin light-chain kinase (MLCK). In the porcine intestinal epithelial cell line IPEC-J2 challenged with ETEC K88, *Limosilactobacillus reuteri* LR1 preserved transepithelial electrical resistance, reduced macromolecular flux, restored claudin-1 and occludin expression, and improved wound closure. These effects were associated with suppression of MLCK signaling [59]. Butyrate can also reinforce epithelial junctions. In weaned piglets, sodium butyrate increased colonic tight-junction protein abundance and reduced permeability and diarrhea [60]. By maintaining junctional continuity during epithelial turnover, these microbial signals restrict persistent stimulation of lamina propria leukocytes.

Microbial exposure also modifies mucosal antimicrobial defenses [61]. Antimicrobial peptides and proteins involved in this response include porcine β-defensins, cathelicidins, lysozyme, and regenerating islet-derived proteins, although their cellular sources differ among intestinal regions and inflammatory conditions [62,63,64]. For example, ETEC F4ac challenge increased ileal PR-39 transcript abundance in both Jinhua and Landrace pigs, with a stronger response in Jinhua pigs [65]. Together with the IAld-associated increase in MUC2-positive cells described above, these findings show that commensal metabolites and enteric pathogens can alter mucus- and peptide-based epithelial defenses.

### 4.3. Regulation of Innate Immune Cells

Microbial material that crosses the epithelium or is actively sampled from the lumen is encountered by intestinal macrophages and DCs. Macrophages phagocytose translocated bacteria and apoptotic epithelial cells, produce cytokines, and contribute to tissue repair. Their phenotypes span a continuum of inflammatory, antimicrobial, regulatory, and reparative states rather than conforming to discrete M1 and M2 categories [66]. Intracellular regulation further determines how macrophages respond to microbial stimulation. In murine macrophage models, methyltransferase-like protein 3 (METTL3) installs m^6^A modifications cotranscriptionally on nascent *Jak1*, *Nlrp3*, and *Il1b* transcripts. Recognition of these modifications by YTH N6-methyladenosine RNA-binding protein 1 (YTHDF1) promotes translation and coordinates with JAK1-dependent transcriptional priming to enhance NLRP3 inflammasome activation and IL-1β production [67]. Whether this METTL3-dependent mechanism operates in porcine intestinal macrophages after weaning remains to be established. Single-cell analysis of the piglet ileum found no significant differences in the proportions of major innate immune-cell populations between suckling and post-weaned piglets. However, macrophages exhibited 176 differentially expressed genes, with enrichment of pathways associated with antigen processing and presentation. Post-weaning samples also showed redistribution of CD103-positive DCs toward the ileal villi and increased predicted interactions between DCs and enterocytes or cytotoxic T lymphocytes [34]. These findings suggest that weaning induces functional and spatial reprogramming of intestinal myeloid cells rather than their simple numerical expansion.

Bacterial extracellular vesicles provide one route by which microbial cargo can reach and modulate myeloid cells without translocation of intact bacteria. In piglets sampled across weaning, single-cell transcriptomic and microbial profiling identified *Bacteroides uniformis* enrichment. In vitro mouse bone marrow-derived macrophages experiments and a murine colitis model then showed that extracellular vesicles from this bacterium promoted pro-inflammatory macrophage polarization and aggravated intestinal inflammation [68]. By contrast, extracellular vesicles produced by *Limosilactobacillus mucosae* isolated from healthy neonatal piglets shifted macrophages away from pro-inflammatory phenotypes and toward repair-associated states. These vesicles attenuated intestinal injury in ETEC K88-challenged mice, and macrophage-depletion experiments indicated that their protective effects were macrophage-dependent [69]. The effects of bacterial vesicles are therefore highly dependent on their microbial origin and molecular cargo.

DCs connect microbial recognition to adaptive mucosal immunity. After acquiring luminal antigens, intestinal DCs can migrate to draining mesenteric lymph nodes or act locally within organized lymphoid tissues. They regulate T-cell and B-cell responses through antigen presentation, costimulatory signals, cytokines, and retinoic acid. In mice, CD103-positive intestinal DCs promote Forkhead box P3 (Foxp3)-positive regulatory T-cell (Treg) differentiation through retinoic acid and transforming growth factor-β [70]. Butyrate also modifies myeloid-cell function through multiple mechanisms. Experiments with human monocyte-derived macrophages, supported by mouse studies, showed that histone deacetylase inhibition by butyrate induces an antimicrobial program in intestinal macrophages [71], whereas activation of G protein-coupled receptor (GPR) 109A in murine colonic macrophages and DCs promotes anti-inflammatory activity and supports the differentiation of regulatory and IL-10-producing T cells [72]. Porcine evidence comes from comparisons of germ-free and specific-pathogen-free piglets, which demonstrate the developmental influence of microbial colonization. The presence of commensal microbiota increases B-cell and T-cell representation in Peyer’s patches and upregulates gene programs associated with antigen processing, B-cell activation and differentiation, germinal-center responses, and IgA isotype switching, particularly in jejunal Peyer’s patches [73]. These findings indicate that microbial products and metabolites shape the signals delivered by intestinal antigen-presenting cells, although most defined DC mechanisms have been established in mice rather than pigs.

Neutrophils provide rapid containment when the epithelial and mononuclear-phagocyte barriers are breached [74]. C-X-C motif chemokine ligand 8 (CXCL8) and other chemotactic signals produced by epithelial and myeloid cells recruit neutrophils, which phagocytose bacteria and release reactive oxygen species, granule proteins, and extracellular traps [75]. Timely recruitment can limit microbial invasion, whereas persistent or excessive activation may damage epithelial cells and delay restitution [76]. In a porcine F18 ETEC challenge model, piglets weaned at 16 or 18 days of age developed more severe diarrhea and barrier injury than piglets weaned at 20 days. The earlier-weaned piglets also exhibited attenuated ileal IL-6 and CXCL8 responses and less neutrophil recruitment, suggesting an impaired capacity to mount an effective early mucosal response [4]. Microbial metabolites can further modulate neutrophil recruitment. In mice, GPR43 contributes to SCFA-dependent regulation of neutrophil trafficking during intestinal inflammation [77]. Effective innate defense therefore requires a sufficiently rapid antimicrobial response followed by timely resolution to prevent persistent tissue injury.

### 4.4. Regulation of Lymphoid Immunity

#### 4.4.1. Type 3 Immunity

Type 3 immunity is mediated primarily by retinoic acid receptor-related orphan receptor γt (RORγt)-expressing ILC3s and Th17 cells, with additional contributions from IL-17-producing γδ T-cell subsets at epithelial barriers [78]. Microbial stimulation of epithelial cells, macrophages, and DCs promotes the production of IL-1β and IL-23, whereas microbiota-derived and dietary aryl hydrocarbon receptor (AhR) ligands act on ILC3s and selected T-cell populations. Depending on the cellular and environmental context, these signals support the production of IL-17A, IL-17F, and IL-22. IL-17 induces epithelial chemokines and granulopoietic factors that promote neutrophil production and recruitment. IL-22 acts predominantly on epithelial cells, enhancing antimicrobial-protein expression, mucus production, cell survival, and tissue repair [37,79]. Porcine studies provide direct evidence that ILC3s participate in early intestinal responses. In the jejunum of suckling piglets, the proportion of ILC3s remained relatively stable from days 1 to 21 after birth but increased markedly on day 28. Steady-state production of ILC3-derived IL-22 did not change significantly over this developmental period. However, acute ETEC exposure increased both ILC3 frequency and IL-22 production within 4 h, indicating rapid activation during bacterial challenge [37]. Complementary evidence has been obtained in a porcine epidemic diarrhea virus (PEDV) model. Oral administration of *Lacticaseibacillus rhamnosus* GG reduced PEDV-associated intestinal injury in piglets, while mechanistic experiments showed that extracellular vesicles derived from this bacterium contained putative AhR-active metabolites and stimulated porcine ILC3 proliferation and IL-22 production in an AhR-dependent manner. In IPEC-J2 and porcine intestinal-organoid coculture systems, ILC3-derived IL-22 activated epithelial signal transducer and activator of transcription 3 (STAT3) signaling, promoted epithelial and organoid growth, increased antimicrobial-gene expression, and reduced PEDV-induced epithelial injury [79]. Porcine epithelial cytokine responses also provide evidence of direct microbial regulation. F4-positive ETEC induced epithelial IL-17C through TLR5, while IL-17C increased pBD-2 and junctional protein expression in porcine intestinal epithelial cells [80].

γδ T cells are particularly abundant in pigs and constitute a major component of the intestinal intraepithelial T-cell compartment during the peri-weaning period [81]. These cells can combine cytotoxic activity, cytokine production, microbial surveillance, and support for epithelial repair, and selected subsets can rapidly produce IL-17. Longitudinal analysis showed that γδ T cells represented a major natural T-cell population in the jejunal epithelium around weaning and declined after weaning as induced CD8αβ-positive αβ T cells accumulated. In piglets weaned at 25 to 28 days of age, this transition occurred within the first 3 days after weaning, whereas in piglets weaned at 18 to 21 days, comparable changes were delayed until days 3 to 7. The earlier-weaned group also exhibited more prolonged restructuring of the jejunal mucosa-associated microbiota [82]. These findings indicate that weaning age influences the temporal coordination of intraepithelial T-cell development and microbial community stabilization.

The clearest causal evidence for microbial induction of Th17 cells comes from mice, in which segmented filamentous bacteria promote intestinal Th17 differentiation [83]. Whether a comparable response to segmented filamentous bacteria or another defined taxon occurs in weaned piglets remains unknown. In piglets, ileal single-cell analysis showed that weaning reduced the relative abundance of Th17 cells but shifted the remaining cells toward a more inflammatory transcriptional state characterized by increased TNF expression [34]. The biological effect of type 3 immunity therefore cannot be inferred from cell abundance alone. Appropriately regulated IL-17 and IL-22 responses support antimicrobial defense and epithelial repair [84,85]. By contrast, sustained IL-1β and IL-23 signaling, persistent recruitment of neutrophils, and prolonged type 3 cytokine production in the context of impaired barrier integrity may maintain mucosal inflammation and exacerbate epithelial injury.

#### 4.4.2. Treg-Mediated Immune Regulation

Foxp3-positive regulatory T (Treg) cells limit excessive immune responses to commensal microorganisms and dietary antigens through several mechanisms, including the production of IL-10 and transforming growth factor-β (TGF-β), cytotoxic T-lymphocyte-associated protein 4 (CTLA-4)-mediated modulation of antigen-presenting cells, and consumption of locally available IL-2. In pigs, peripheral-blood CD4^+^CD25^high^ T cells express Foxp3 and suppress the proliferation of responder T cells in vitro. This suppressive activity is partly dependent on direct cell contact and competition for IL-2 [86]. However, the development and function of intestinal Treg cells during the post-weaning period remain poorly characterized in pigs. Evidence that the microbiota promotes peripheral Treg differentiation has been obtained primarily from mouse studies. Colonization with indigenous bacterial species formerly assigned to *Clostridium* clusters IV and XIVa promoted the accumulation of Foxp3-positive Treg cells in the colon [87]. Microbiota-derived butyrate also enhanced colonic Treg differentiation by inhibiting histone deacetylases and increasing histone acetylation at regulatory regions of the *Foxp3* locus. More broadly, SCFAs increased the size and suppressive capacity of the colonic Treg pool through mechanisms involving the SCFA receptor GPR43 [88,89]. These findings establish plausible pathways through which microbial fermentation may promote mucosal tolerance, although their operation in the intestine of weaned piglets has not been demonstrated directly.

Peripheral Treg and Th17 differentiation are closely related but are not governed by a simple binary switch. In mouse models, TGF-β promotes Foxp3 induction under non-inflammatory conditions, whereas the presence of IL-6 or IL-21 favors Th17 differentiation. IL-1β can reinforce the Th17 program, while IL-23 primarily supports the expansion, stability, and inflammatory potential of differentiated Th17 cells. Retinoic acid generally favors Treg differentiation and restrains Th17 development. Porcine studies indicate that weaning alters the abundance and transcriptional state of ileal Th17 cells, whereas comparisons between germ-free and specific-pathogen-free piglets demonstrate that microbial colonization promotes the maturation of antigen-presenting and lymphoid compartments in Peyer’s patches [34,73]. These observations support an association between microbial colonization and mucosal lymphocyte development but do not establish direct microbial induction of Treg cells after weaning. Consequently, the functional balance between type 3 immunity and Treg-mediated restraint cannot be inferred from total CD4^+^ T-cell abundance alone.

#### 4.4.3. B Cells and Mucosal IgA Responses

Microbial antigens enter Peyer’s patches through M cells or are delivered by mucosal antigen-presenting cells. T cell-dependent IgA responses develop within germinal centers, where signals from T follicular helper cells induce activation-induced cytidine deaminase and support IgA class-switch recombination, somatic hypermutation, affinity maturation, and the generation of plasma and memory B cells. T cell-independent responses can arise more rapidly when microbial TLR signals act in combination with B-cell-activating factor (BAFF), a proliferation-inducing ligand (APRIL), retinoic acid, and TGF-β derived from epithelial or myeloid cells, although these mechanisms have been defined primarily in mice and humans [90]. In pigs, comparison of germ-free and specific-pathogen-free animals showed that the absence of microbial colonization resulted in hypoplastic Peyer’s patches and reduced B- and T-cell compartments. Colonization was associated with the induction of transcriptional programs related to antigen processing, germinal-center formation, B-cell proliferation and differentiation, and IgA class switching [73]. These findings identify microbial colonization as an important stimulus for the maturation of porcine Peyer’s patches and their B-cell compartments.

Lamina propria plasma cells secrete J chain-containing dimeric IgA, which binds to the polymeric immunoglobulin receptor (pIgR) on the basolateral surface of epithelial cells. Following transcytosis and cleavage of pIgR, IgA is released into the lumen as sIgA [91,92]. Microbial metabolites can also regulate this response [93]. In mice, acetate promoted intestinal IgA production through a GPR43-dependent and T cell-independent mechanism. Complementary coculture experiments indicated that GPR43 signaling in dendritic cells increased *Aldh1a2* expression and retinoic acid synthesis, thereby promoting IgA class switching in B cells [94]. Whether this acetate–GPR43 pathway operates in weaned piglets has not been demonstrated. Together with the porcine colonization studies described above, these findings indicate that microbial exposure supports B-cell maturation, IgA class switching, and mucosal IgA production. The subsequent effects of secreted IgA on microbial colonization and spatial organization are discussed in Section 5.2.

### 4.5. Microbial Metabolites as Mediators of Mucosal Immunity

#### 4.5.1. Short-Chain Fatty Acids

Acetate, propionate, and butyrate are produced through microbial fermentation of non-digestible carbohydrates, and their intestinal concentrations depend on substrate availability, microbial cross-feeding, transit time, and host absorption [95,96]. SCFAs activate receptors with overlapping ligand specificity, principally free fatty acid receptor (FFAR) 2/GPR43, FFAR3/GPR41, and hydroxycarboxylic acid receptor 2 (HCAR2)/GPR109A. After cellular uptake, butyrate and propionate can also inhibit histone deacetylases. Butyrate serves as an oxidative energy source for colonocytes and regulates epithelial proliferation, differentiation, tight-junction assembly, and antimicrobial gene expression. In piglets, post-weaning changes in jejunal butyrate concentration and GPR43 expression occur in parallel with changes in epithelial proliferation and apoptosis [57]. Dietary sodium butyrate increased ileal and colonic tight-junction protein abundance and reduced intestinal permeability and diarrhea in weaned piglets, whereas involvement of GPR109A–Akt signaling was demonstrated in Caco-2 cells [60].

SCFAs also regulate myeloid and lymphoid cells. Experiments with human monocyte-derived macrophages, supported by mouse studies, butyrate induces antimicrobial macrophage programs through histone deacetylase inhibition [71], while GPR109A signaling in macrophages and DCs promotes IL-10 production and Treg differentiation [72]. Butyrate and other SCFAs promote the accumulation and function of colonic Treg cells through receptor-dependent and epigenetic mechanisms [88,89], and GPR43 regulates neutrophil recruitment during inflammation [77]. Acetate also promotes T cell-independent IgA production by stimulating GPR43-dependent retinoic acid synthesis in DCs [94]. These effects vary with SCFA identity and concentration, receptor distribution, cellular metabolism, and the inflammatory state of the mucosa. Equivalent cell-specific effects of SCFAs have not yet been demonstrated in porcine intestinal immune cells.

#### 4.5.2. Tryptophan-Derived Metabolites

Gut bacteria convert tryptophan into indole and multiple derivatives, including IAld, indole-3-lactic acid, indole-3-acetic acid, and indole-3-propionic acid [97]. Several of these metabolites activate AhR in epithelial cells, ILC3s, and T cells, whereas indole-3-propionic acid can activate the pregnane X receptor (PXR) in intestinal epithelial cells. In mice, IAld–AhR signaling induces IL-22 production and promotes mucosal antimicrobial defense [98], while indole-3-propionic acid–PXR signaling reduces epithelial permeability and restrains TLR4-associated inflammation [99]. AhR and PXR therefore translate microbial tryptophan metabolism into epithelial and mucosal immune responses.

Porcine studies support both epithelial and lymphoid targets of these metabolites. Fecal IAld concentrations and the abundance of genes involved in microbial tryptophan catabolism were lower in diarrheal than in healthy weaned piglets [58]. Dietary IAld promoted intestinal stem-cell expansion, epithelial differentiation, MUC2 expression, and tight-junction protein abundance in vivo, while also increasing the growth of porcine intestinal organoids. These responses were accompanied by increased AhR target-gene expression and Wnt/β-catenin signaling [58]. In PEDV-challenged piglets, oral administration of *Lacticaseibacillus rhamnosus* GG reduced diarrhea, intestinal injury, and viral load and altered intestinal ILC3 and IL-22 responses. Mechanistic experiments showed that LGG-derived extracellular vesicles containing putative AhR ligands stimulated porcine ILC3 proliferation and IL-22 production through AhR in vitro [79]. Subsequent coculture experiments linked ILC3-derived IL-22 to STAT3 activation, epithelial proliferation, organoid growth, and reduced PEDV-induced epithelial injury [79]. Microbial tryptophan metabolites may therefore link luminal nutrient metabolism to epithelial renewal and IL-22-mediated mucosal protection.

#### 4.5.3. Bile Acids and Other Microbial Metabolites

Conjugated primary bile acids entering the intestine are deconjugated and subsequently 7α-dehydroxylated, epimerized, or oxidized by microbial enzymes, generating a chemically diverse pool of secondary bile acids. Selected primary and secondary bile acids activate the nuclear receptor farnesoid X receptor (FXR)/nuclear receptor subfamily 1 group H member 4 (NR1H4) and the membrane receptor Takeda G protein-coupled receptor 5 (TGR5)/G protein-coupled bile acid receptor 1 (GPBAR1) in epithelial, hepatic, and immune cells, thereby regulating epithelial metabolism, antimicrobial defense, and cytokine production [100]. In weaned piglets, supplementation with *Lactiplantibacillus plantarum* altered fecal bacterial genera associated with bile-acid transformation and reduced the total lithocholic acid concentration in the ileum [101]. More detailed lymphocyte mechanisms have been defined in mice. 3-Oxo-lithocholic acid directly inhibits RORγt and Th17 differentiation, whereas isoallo-lithocholic acid promotes Foxp3-positive Treg differentiation through mitochondrial reactive oxygen species [102]. Microbial transformation of the bile-acid pool can therefore influence epithelial signaling and the balance between Th17 and Treg responses [103]. Whether the same bile-acid-dependent control of Th17/Treg balance occurs in weaned piglets remains unknown; current porcine evidence shows altered bile-acid profiles but does not establish a causal lymphocyte mechanism.

Lactate, succinate, and polyamines provide additional routes of microbial regulation. In porcine jejunal organoids, *Lactobacillus amylovorus*-derived lactate promoted intestinal stem-cell proliferation through GPR81-dependent activation of Wnt/β-catenin signaling [104]. Evidence from Crohn’s disease tissue, human fibroblasts, and murine colitis and fibrosis models indicates that excessive succinate signaling through succinate receptor 1 (SUCNR1) can amplify intestinal inflammation [105]. Diarrheal weanling piglets exhibited increased microbiota-derived succinate. Fecal microbiota transplantation from healthy donors reduced succinate accumulation and diarrhea, whereas rectal succinate administration induced diarrhea in healthy piglets [106]. Polyamines originate from dietary, host, and microbial amino-acid metabolism and support epithelial proliferation, migration, and junctional integrity. Exogenous spermine increased ileal tight-junction protein expression and antioxidant capacity and altered the ileal metabolic profile in piglets [107]. Other products of amino-acid fermentation, including ammonia, biogenic amines, phenolic compounds, and sulfur-containing metabolites, may impair epithelial and immune homeostasis when their production exceeds local utilization and clearance [108]. Thus, the effects of these metabolites depend on their concentration, site of production, and capacity for host or microbial utilization.

Taken together, microbial structural components and metabolites influence the post-weaning mucosa at several levels, from epithelial renewal and barrier function to innate and adaptive immune responses. The epithelial and immune products generated in response to these signals can, in turn, alter the conditions encountered by intestinal microorganisms. Their effects on microbial localization, persistence, and competitive fitness are considered in the following section.

## 5. Mucosal Immune Regulation of the Gut Microbiota

**Figure 3:**
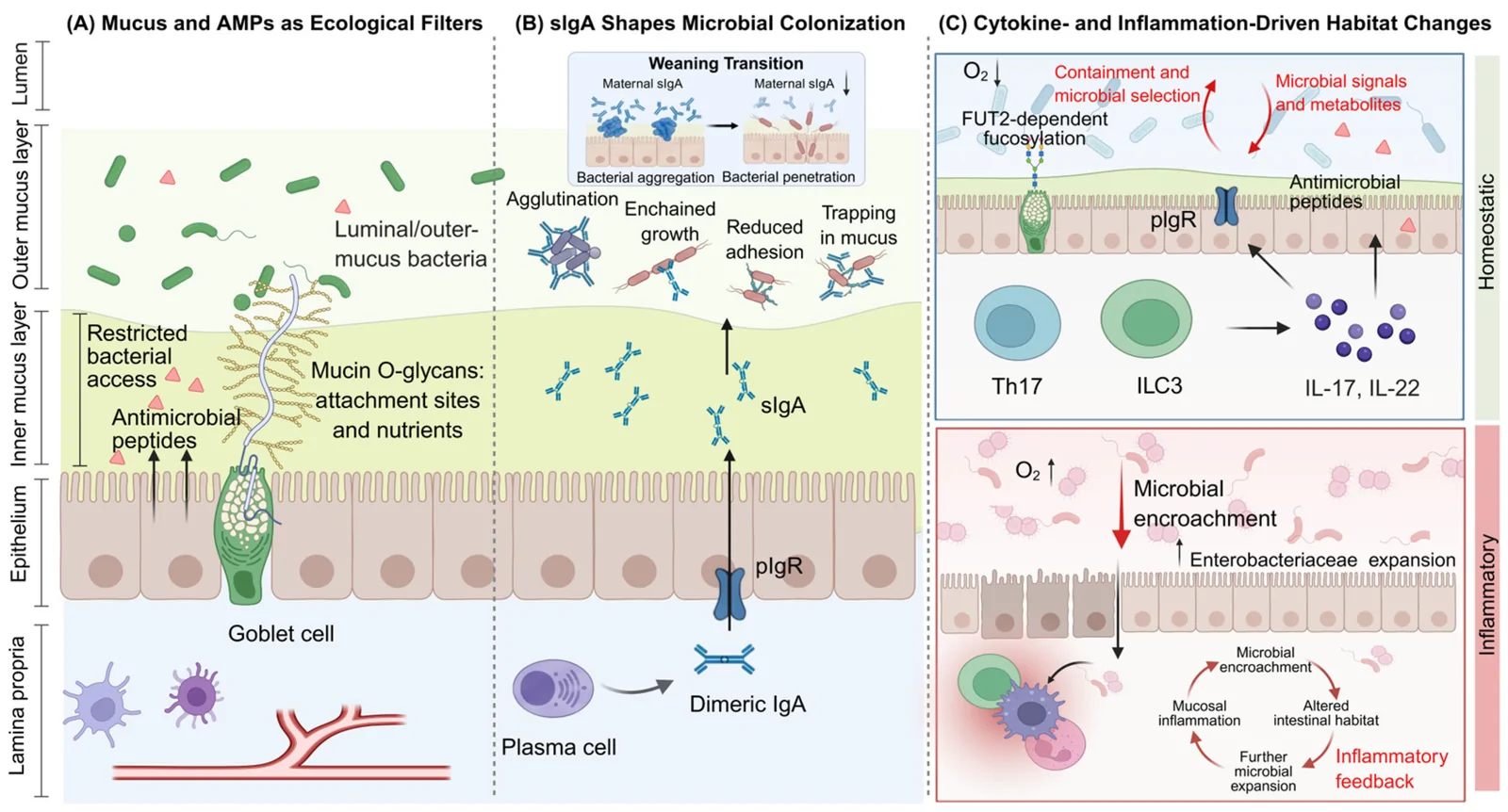
Mucosal immune regulation of microbial localization and ecological selection in weaned piglets. (**A**) Mucus and antimicrobial peptides retain bacteria in the lumen or outer mucus layer and restrict their access to the epithelium. Mucin O-glycans also provide attachment sites and nutrients for selected bacteria. (**B**) sIgA, transported by the pIgR, regulates bacterial aggregation, enchained growth, adhesion, and trapping within mucus. Loss of maternal sIgA at weaning can increase bacterial penetration. (**C**) IL-17 and IL-22 regulate epithelial fucosylation, pIgR expression, and antimicrobial activity. During inflammation, increased oxygen availability and barrier disruption favor Enterobacteriaceae expansion and microbial encroachment, which can further amplify mucosal inflammation. The IL-17/IL-22-dependent and inflammation-driven ecological mechanisms in panel C are based mainly on mouse studies; comparable host-driven changes in microbial ecology have not been established directly in weaned piglets. Abbreviations: AMPs, antimicrobial peptides; FUT2, fucosyltransferase 2; IgA, immunoglobulin A; ILC3, group 3 innate lymphoid cell; IL-17, interleukin-17; IL-22, interleukin-22; O_2_, molecular oxygen; pIgR, polymeric immunoglobulin receptor; sIgA, secretory immunoglobulin A; Th17, T helper 17 cell.

The epithelial and immune responses described in the previous section also influence the microorganisms exposed to them. The mucosal immune system is a major determinant of intestinal microbial ecology. By regulating microbial access to the epithelial surface, the availability of host-derived substrates, and local physicochemical conditions, it shapes microbial persistence and spatial organization without eliminating luminal communities [25,109]. Mucus and antimicrobial molecules establish region-specific barriers, sIgA imposes antibody-mediated selection, and cytokines modify epithelial secretory and metabolic programs [14,110]. These effects are particularly important after weaning, when withdrawal of milk-derived protection and ongoing mucosal maturation coincide with rapid microbial succession [3,21]. This section therefore focuses on the consequences of these host responses for microbial localization, persistence, and colonization resistance, rather than on the pathways by which the responses are induced. Figure 3 summarizes these effects across the lumen–mucus–epithelium axis. Mucus and antimicrobial peptides restrict bacterial access to the epithelial surface (Figure 3A), sIgA regulates bacterial aggregation, adhesion, and retention within mucus (Figure 3B), and cytokine- and inflammation-dependent changes in epithelial defense and metabolism reshape the microbial habitat (Figure 3C).

### 5.1. Mucus and Antimicrobial Peptides as Ecological Filters

The mucus layer creates a physical gradient between the luminal microbiota and the epithelial surface (Figure 3A). In the small intestine, the mucus gel is relatively penetrable, and microbial separation also depends on luminal flow and antimicrobial activity near the epithelium. In mice, polymerized MUC2 in the colon forms a dense inner layer containing few bacteria and a looser outer layer colonized by mucus-associated microorganisms [111]. Continuous mucin secretion, outward expansion, and shedding limit sustained bacterial contact with epithelial cells while retaining a large microbial population in the lumen. In piglets, goblet-cell abundance, mucin expression, and other barrier features follow distinct postnatal developmental patterns in the small and large intestine, indicating segment-specific maturation of the spatial constraints on microbial colonization [112]. Evidence from diarrheal piglets illustrates the ecological consequences of weakened mucus containment. Reduced goblet-cell numbers and MUC2 expression, thinning of the colonic mucus layer, and a shift from acidic and extended O-glycans toward truncated glycan structures were accompanied by bacterial encroachment, impaired tight-junction integrity, and mucosal inflammation [113].

Mucin glycans add nutritional selectivity to this physical barrier. Mucin O-glycans contain fucose, galactose, N-acetylglucosamine, N-acetylgalactosamine, and sialic acid, which provide attachment sites and carbon sources for bacteria possessing the appropriate adhesins, glycosidases, and polysaccharide-utilization systems. In gnotobiotic mice, the capacity to utilize mucin O-glycans enhanced the intestinal persistence and transmission of *Bacteroides thetaiotaomicron*, while released monosaccharides may also support cross-feeding among community members [114]. Excessive mucin degradation, however, can weaken microbial containment. In gnotobiotic mice, dietary fiber deprivation redirected microbial metabolism toward host mucin glycans, expanded mucus-degrading populations, eroded the colonic mucus barrier, and increased susceptibility to *Citrobacter rodentium* infection [115]. Host glycosylation may also contribute to microbial selection in pigs. Variants in *MUC4* and *FUT1* were associated with jejunal fucose- and galactose-binding patterns, *REG3G* and *TFF3* expression, and mucosa-associated microbiota composition in post-weaning pigs [116]. Mucin production and glycosylation therefore influence both substrate availability near the mucosa and the microorganisms capable of occupying this habitat.

Antimicrobial peptides and proteins reinforce this spatial compartmentalization. Defensins, cathelicidins, REG-family lectins, lysozyme, and related molecules are released at or near the epithelial surface, including crypt regions, where their effects vary with bacterial envelope structure, protease resistance, and proximity to the site of secretion. In mice, altered α-defensin activity changed small-intestinal community composition with little effect on total bacterial abundance, whereas transgenic expression of human defensin alpha 5 (DEFA5) markedly reduced segmented filamentous bacteria and the associated mucosal Th17 response [117]. REGIIIγ provides a direct example of spatial control in mice: this peptidoglycan-binding lectin maintains an approximately 50-μm separation between bacteria and the small-intestinal epithelium, whereas its absence increases bacterial contact with the mucosal surface [118]. Porcine epithelial defense programs also change across the weaning period. Compared with 21-day-old suckling piglets, 35-day-old weaned piglets showed higher expression of *REG3G*, *PIGR*, *DUOX2*, and *NOX1* in jejunal crypts [119]. Under homeostatic conditions, this antimicrobial repertoire restricts bacterial encroachment and shapes the microorganisms that approach the mucosa without indiscriminately reducing luminal bacterial biomass.

### 5.2. sIgA Shapes Microbial Colonization

Once released into the intestinal lumen, sIgA adds antibody specificity to the physicochemical barrier formed by mucus (Figure 3B). Its secretory component increases proteolytic resistance and promotes retention within the mucus layer. Binding to bacterial surface antigens, flagella, and adhesins, as well as soluble toxins, can inhibit motility, epithelial attachment, and toxin activity. Coated microorganisms may also be trapped within mucus and removed by intestinal flow, a process broadly termed immune exclusion [120]. The multivalency of sIgA further supports bacterial agglutination. In mice, high-avidity IgA can cross-link adjacent daughter cells during bacterial division and prevent their separation, generating clonal aggregates that restrict dissemination. This process, termed enchained growth, accelerated the clearance and limited the transmission of enteric bacteria [121].

IgA coating does not invariably result in bacterial elimination. In mice, natural polyreactive IgA recognizes a broad but defined subset of the resident microbiota and provides basal containment of commensal organisms [122]. Some bacteria can also exploit IgA to stabilize their mucosal niche. In mice, *Bacteroides fragilis* modifies its capsular surface to promote IgA coating, which facilitates mucosal association, bacterial aggregation, and resistance to displacement by incoming strains [123]. IgA binding can also alter bacterial gene expression and metabolism. In a gnotobiotic mouse model, IgA binding to *Bacteroides thetaiotaomicron* regulated mucus-associated functional factors and polysaccharide-utilization loci, thereby influencing its mucosal fitness and metabolic interactions with other community members [124]. The outcome of IgA coating therefore depends on antibody affinity and epitope specificity, together with the surface architecture and physiological state of the bacterium. IgA may exclude adhesive or rapidly expanding organisms while retaining selected commensals within mucus and supporting community stability. Whether these strain-specific IgA effects are conserved in pigs has not yet been established. Evidence involving piglets further supports the role of IgA in microbial organization. A study combining observations in weaned piglets with mechanistic experiments in mice showed that CD138-positive plasma cell-derived IgA confined *Bacteroides uniformis* to a defined luminal niche, whereas disruption of the IgA response altered its spatial distribution and aggravated intestinal inflammation [125]. Oral administration of *Akkermansia muciniphila* to nursing piglets also increased intestinal sIgA concentrations and altered the composition of the sIgA-coated bacterial community after early weaning [126].

Maternal sIgA also contributes to microbial containment and protection of the neonatal intestine. In germ-free neonatal piglets, oral challenge with *Escherichia coli* O55 preincubated with human colostrum- and milk-derived sIgA improved intestinal histology, clinical condition, and survival. Removal of sIgA N-glycans reduced this protection without abolishing bacterial agglutination, indicating that glycan-mediated interactions contribute to protection beyond antigen-dependent cross-linking [127]. In an ex vivo analysis of intestinal mucus from suckling and weaned piglets, motile *E. coli* penetrated more than 100 μm into mucus collected after weaning, whereas suckling-piglet mucus promoted bacterial aggregation and restricted penetration. Addition of suckling-mucus supernatant or purified human milk IgA restored aggregation and reduced bacterial entry into weaned-piglet mucus [128]. These findings suggest that the loss of maternal luminal IgA at weaning contributes to reduced bacterial aggregation and greater microbial penetration of the mucus compartment.

### 5.3. Cytokine- and Inflammation-Driven Changes in the Microbial Habitat

Cytokines produced during mucosal immune responses can subsequently reshape microbial ecology by altering epithelial antimicrobial activity, glycosylation, micronutrient availability, and local physicochemical conditions (Figure 3C). In mice, epithelial IL-17 receptor signaling induced α-defensins, pIgR, and NADPH oxidase 1, whereas disruption of this signaling reduced these defense programs and increased the abundance of segmented filamentous bacteria [129]. Murine studies further show that IL-22 induces epithelial antimicrobial proteins and Fut2-dependent epithelial fucosylation. Cleavage of luminal fucosylated glycans by bacterial fucosidases releases fucose, which can modify microbial metabolic and virulence-associated programs [84,130]. Cytokine-induced defenses can nevertheless affect closely related organisms differently. During *Salmonella* Typhimurium infection in mice, IL-22-dependent induction of lipocalin-2 and calprotectin suppressed susceptible commensal Enterobacteriaceae, whereas *Salmonella* resisted these nutrient-withholding mechanisms and gained a competitive advantage [131]. Cytokine signaling can therefore shape microbial access to the mucosal surface, host glycans, and essential micronutrients. Whether IL-17- and IL-22-dependent ecological selection operates similarly in weaned piglets has not been tested directly.

In mouse models, inflammation produces broader changes in the intestinal habitat and can promote Enterobacteriaceae expansion [132]. Microbial metabolites can also influence microbial competition indirectly through host epithelial metabolism. In mice, microbiota-derived butyrate activates epithelial peroxisome proliferator-activated receptor gamma (PPAR-γ) signaling and supports mitochondrial β-oxidation. The resulting oxygen consumption maintains epithelial hypoxia and limits oxygen diffusion toward the colonic lumen [133]. Other murine experiments showed that depletion of butyrate-producing obligate anaerobes reduced colonocyte oxygen consumption and increased oxygen availability at the mucosal surface, favoring the expansion of facultative anaerobes through aerobic respiration [134]. Inflammation also generates alternative electron acceptors. In inflamed mice, inducible nitric oxide synthase-derived nitric oxide contributes to the formation of host-derived nitrate, and nitrate respiration provides *E. coli* with a growth advantage over bacteria that rely predominantly on fermentation [135]. Tissue injury and accelerated epithelial turnover further increase the availability of host-derived nutrients. During intestinal inflammation in mice, *Salmonella* Typhimurium used host-derived ethanolamine as a carbon source through tetrathionate-supported respiration, thereby enhancing its competitive fitness [136]. Taken together, these murine studies show that increased oxygen availability, alternative electron acceptors, and host-derived substrates favor organisms capable of respiratory growth and rapid exploitation of the inflamed mucosal environment.

These ecological mechanisms may also operate after weaning, although changes in luminal oxygen and nitrate have not been measured directly in piglets. Colonic transcriptional responses within one day after weaning include enrichment of TLR-, NOD-like receptor-, cytokine-, interferon-, bacterial-defense-, and oxidoreductase-related pathways, together with transient barrier impairment [40]. These findings are consistent with changes in the mucosal environment but do not establish which respiratory substrates become available to the microbiota. When mucus-mediated separation, antibody containment, and metabolic control are weakened, inflammation-adapted organisms may gain a competitive advantage and further aggravate mucosal injury. The consequences of this loss of coordination are considered in Section 6.

## 6. Dysregulation of Microbiota–Mucosal Immune Interactions in Weaned Piglets

Post-weaning microbial succession is a physiological process, but it may become maladaptive when microbial functional adaptation, epithelial repair, and mucosal immune maturation fail to proceed in a coordinated manner [3,21]. Persistent disturbances in microbial function and localization, barrier integrity, and immune responsiveness can then reinforce one another, increasing susceptibility to enteric infection, diarrhea, and impaired growth [19].

### 6.1. Destabilization of the Microbial–Immune Interface

The marked reduction in feed intake immediately after weaning is an early driver of intestinal instability. Reduced nutrient delivery to the epithelium and microbiota contributes to villus atrophy and impaired barrier function, while the microbial community must simultaneously adapt to rapidly changing dietary substrates [2,42]. Milk withdrawal also removes maternal immunoglobulins and other bioactive factors that support mucosal homeostasis. The loss of maternal IgA-mediated bacterial aggregation may further weaken spatial containment after weaning [128].

Maternal separation, social mixing, altered housing, pathogen exposure, and antimicrobial treatment impose additional disturbances during the same period. A longitudinal study showed that the ileal microbiota was initially destabilized following the introduction of solid feed and displayed substantial inter-individual variability; a high-protein diet was also associated with lower community stability [137]. Weaning further alters microbial metabolic output and functional pathways involved in carbohydrate, amino acid, and SCFA metabolism [27]. Dysregulation is therefore more appropriately characterized by impaired microbial function, spatial containment, and ecological resilience than by any single change in richness or taxonomic abundance. Disrupted cross-feeding, insufficient production of barrier-supportive metabolites, increased proteolytic fermentation, and delayed restoration of colonization resistance may prolong the exposure of the immature mucosa to unfavorable microbial signals.

### 6.2. Barrier Dysfunction and Exaggerated Mucosal Inflammation

Reduced mucus-mediated exclusion and disruption of tight junctions increase mucosal exposure to microbial ligands. Early weaning reduced transepithelial resistance, altered junctional protein expression, and activated p38, JNK, and ERK1/2 signaling in the porcine intestine [33]. These barrier changes coincided with increased expression of inflammatory cytokines, linking epithelial injury with mucosal immune activation [138]. Greater access of microbial products to epithelial and lamina propria receptors promotes cytokine and chemokine production and the recruitment of monocytes and neutrophils [62,139]. Although these cells contribute to microbial containment, their cytokines, reactive oxygen species, and proteases can aggravate epithelial injury and permit further penetration of microbial products [74].

Inflammasome activation can amplify this feedback. Piglets with post-weaning diarrhea exhibited increased NLRP3 activation and pyroptosis, while experimental infection with ETEC K88 or *Clostridium perfringens* disrupted intestinal integrity and increased serum IL-1β and IL-18 concentrations and diarrhea incidence [140]. Altered lymphocyte responses may also contribute. Single-cell analysis of the ileum identified a lower relative abundance of Th17 cells after weaning but a shift of the remaining population toward a more inflammatory transcriptional state. CD8-positive, granzyme B-positive (CD8^+^GZMB^+^) cytotoxic T cells were also enriched, and computational analyses linked these cells to epithelial mitochondrial dysfunction and apoptosis [34]. Mucosal dysregulation, however, does not involve a uniform increase in all inflammatory responses. Following F18 ETEC challenge, piglets weaned at 16 or 18 days developed more severe barrier injury and diarrhea than those weaned at 20 days, despite weaker IL-6 and IL-8 responses and reduced neutrophil recruitment; ileal TNF-α was selectively increased in piglets weaned at 16 days [4]. Tissue-damaging inflammation can therefore coexist with inadequate pathogen-directed defense, allowing microbial exposure and epithelial injury to persist.

### 6.3. Loss of Colonization Resistance and Enteric Infection

Colonization resistance depends on competition for nutrients and attachment sites, inhibitory microbial products, and intact mucus, antimicrobial peptide, and sIgA defenses. Post-weaning instability may weaken these mechanisms, although ETEC infection additionally requires host susceptibility to F4 or F18 fimbrial adhesion and an appropriate enterotoxin repertoire [141]. Following attachment, ETEC colonizes the small-intestinal surface and may produce LT, STa, and/or STb, which disrupt epithelial ion transport and induce net electrolyte and water secretion [141]. The resulting fluid loss and infection-associated epithelial stress may impair barrier function and increase inflammatory signaling, further altering the microbial habitat.

Dietary conditions can modify this sequence. In weaned pigs exposed to ETEC, a high-protein diet produced the greatest disturbance of ileal community structure and stability and was associated with approximately tenfold higher small-intestinal ETEC counts on day 5 post-inoculation than a low-protein diet [142]. Other pathogens initiate disruption through different mechanisms. Virulent *Salmonella* Typhimurium induced intestinal inflammation and reduced several SCFA-producing bacterial populations in piglets [143], whereas *C. perfringens* combined toxin-mediated epithelial injury with inflammasome activation [140]. PEDV directly damaged villous enterocytes and altered Paneth-cell lysozyme expression, villous and Peyer’s patch M-cell abundance, and epithelial pIgR expression in weaned pigs [144]. Despite their distinct pathogenic mechanisms, these infections converge on epithelial injury, impaired microbial containment, and further erosion of colonization resistance.

### 6.4. Post-Weaning Diarrhea and Growth Impairment

Post-weaning diarrhea develops when intestinal secretion and luminal osmotic load exceed absorptive capacity. ETEC enterotoxins stimulate electrolyte and water secretion, whereas villus atrophy and loss of mature absorptive enterocytes impair brush-border digestion and nutrient-coupled fluid absorption [2,141]. Tight-junction disruption increases paracellular permeability and may promote passive fluid loss under secretory conditions, while inflammatory mediators alter epithelial transport and intestinal motility. Accelerated transit further restricts digestion and absorption, leaving osmotically active nutrients available for altered distal fermentation. Post-weaning diarrhea therefore reflects the combined effects of secretion, malabsorption, increased permeability, altered motility, microbial metabolism, and pathogen expansion [145].

These disturbances also restrict growth. Reduced feed intake limits nutrient supply, while diarrhea, villus injury, and accelerated transit decrease nutrient digestion and retention. Epithelial repair and immune activation further redirect energy and amino acids away from tissue deposition. In an F18 ETEC challenge model, infected pigs had lower final body weight, average daily gain, and average daily feed intake, whereas gain-to-feed ratio remained unchanged because feed intake and weight gain declined proportionally [146]. Earlier weaning was also associated with greater short-term growth depression following F18 ETEC challenge [4]. Associations between microbial profiles and growth may therefore reflect the combined influences of diet, feed intake, epithelial function, inflammation, and pathogen burden and should not be interpreted as direct effects of individual taxa without functional validation. Consistent with this complexity, one longitudinal study found no detectable relationship between ileal community composition and average daily gain during the early post-weaning period [137]. Prolonged intestinal dysfunction can delay epithelial repair and microbial stabilization, impair nursery growth, and increase susceptibility to subsequent enteric disease.

## 7. Nutritional Modulation of Microbiota–Mucosal Immune Interactions

**Figure 4:**
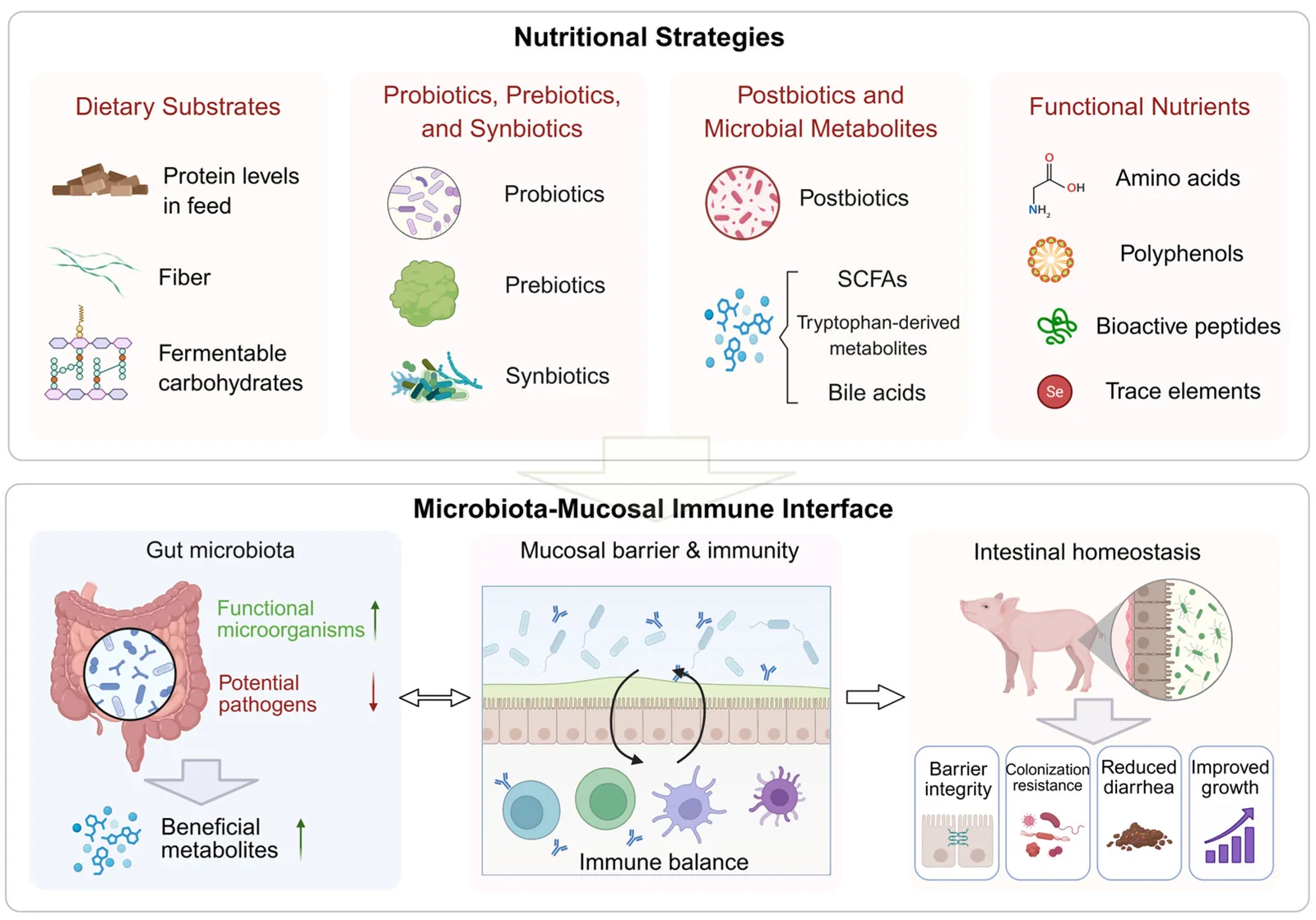
Nutritional modulation of microbiota–mucosal immune interactions in weaned piglets. Dietary substrates, microbial modulators, postbiotics, and functional nutrients regulate gut microbiota and microbial metabolism, thereby supporting mucosal barrier integrity and immune balance. These effects may enhance colonization resistance, reduce intestinal inflammation and diarrhea, and improve growth.

Nutrition is a major determinant of microbiota–mucosal immune interactions during the post-weaning period. It shapes the quantity and composition of substrates available for microbial utilization while supplying nutrients required for epithelial renewal, mucus synthesis, and immune function [147,148]. These processes are particularly interdependent after weaning, when feed intake, digestive capacity, microbial fermentation, and mucosal maturation change rapidly and concurrently [21]. As summarized in Figure 4, nutritional strategies include balanced substrate supply; probiotics, prebiotics, and synbiotics; postbiotics and microbial metabolites; and functional nutrients and bioactive compounds. These interventions may modify microbial composition and metabolism or act directly on host tissues, thereby influencing barrier integrity, mucosal immune regulation, colonization resistance, diarrhea, and growth [48,149].

### 7.1. Dietary Substrate Supply and Microbial Metabolism

Protein that escapes digestion in the small intestine provides carbon and nitrogen for distal intestinal microorganisms. Proteolytic fermentation generates ammonia, branched-chain fatty acids, biogenic amines, phenolic and sulfur-containing compounds, and diverse indole derivatives. Their biological activities differ, but excessive exposure to ammonia, amines, phenols, p-cresol, and hydrogen sulfide can disrupt epithelial metabolism and promote inflammatory responses. In newly weaned pigs, higher dietary protein or greater fermentable protein availability has been associated with reduced ileal community stability, enrichment of potential pathogens, and increased abundance of GH33 exosialidases involved in host-glycan utilization [137,150]. Reducing crude protein while maintaining an adequate profile of standardized ileal digestible amino acids can decrease the amount of fermentable nitrogen entering the hindgut without restricting the amino acid supply required for growth and mucosal protein synthesis.

Fermentable carbohydrates shift microbial metabolism toward saccharolysis, promote the incorporation of nitrogen into bacterial biomass, and increase SCFA production. These effects depend on fiber solubility, viscosity, fermentability, particle size, and accessibility to microbial enzymes. Fermentable fiber attenuated protein-induced changes in colonic microbial ecology in piglets, although it did not reverse the accompanying mucosal responses [151]. A soluble, highly fermentable fiber combined with carbohydrases improved barrier-associated indices and growth during F18 ETEC challenge [152]. Resistant potato starch increased anaerobic fermenters and butyrate concentrations and altered cecal markers associated with barrier function and immune regulation [153]. Dietary fiber also attenuated LPS-induced barrier injury in association with changes in microbial composition and SCFA profiles [154]. Appropriate fermentable carbohydrates can therefore increase the availability of acetate, propionate, and butyrate to epithelial and immune cells, with butyrate also serving as an important oxidative fuel for colonocytes [155,156].

Protein and carbohydrate supply should be considered jointly because fermentable carbohydrates influence whether undigested nitrogen is incorporated into microbial biomass or converted into potentially harmful metabolites [150,157]. In a recent study, an 18% crude-protein diet supplemented with 0.2% isomalto-oligosaccharide reduced diarrhea and fecal indole and skatole concentrations, increased intestinal ZO-1 expression, and maintained growth in weaned piglets; *Bifidobacterium* abundance also showed an increasing trend [158]. These findings are consistent with reduced aromatic amino acid fermentation and improved barrier function.

Translating these nutrient-level relationships into practical diets requires attention to the feed ingredients that provide the substrates. Wheat- and barley-based diets produce different ileal and fecal microbial profiles in pigs, even when their analyzed non-starch polysaccharide contents are similar, indicating that cereal source can influence microbial ecology beyond the total fiber concentration [159]. Oat bran increased the abundance of *Prevotella*, *Butyricicoccus*, and *Catenibacterium* and reduced the expression of IL-8, NF-κB, and TNF-α in the hindgut of growing pigs [160]. High-amylose maize starch likewise shifted the cecal microbiota of weaned piglets, increasing *Lactobacillus* and *Terrisporobacter* while decreasing *Streptococcus* [161]. Protein ingredients also differ in their intestinal effects. Soybean glycinin and β-conglycinin can induce endoplasmic reticulum stress, reduce tight junction proteins and MUC2 expression, promote epithelial apoptosis, and alter microbial composition in weaned piglets [162]. Heating, microbial fermentation, and enzymatic hydrolysis of soybean meal modify its antinutritional factors, nutrient digestibility, and effects on the intestinal microbiota [163]. Feed structure and chemical characteristics provide an additional level of regulation. Particle size and pelleting can affect intestinal mucin distribution and bacterial adhesion [164], whereas the acid-binding capacity of the complete diet influences the gastric environment, growth, and fecal consistency of newly weaned pigs [165]. Thus, crude protein and crude fiber concentrations alone do not fully describe the likely intestinal response; ingredient source, processing method, fermentability, particle size, and acid-binding capacity are also relevant. Loewenstein et al. [166] reported this feeding program for undocked pigs on one farm and listed only the major ingredients. It nevertheless illustrates how cereal source, protein supply, fiber content, and amino acid adequacy can be considered together when formulating post-weaning diets.

### 7.2. Probiotics, Prebiotics, and Synbiotics

Probiotics are live microorganisms that, when administered in adequate amounts, confer a health benefit on the host; prebiotics are substrates selectively utilized by host microorganisms that confer a health benefit; and synbiotics are mixtures comprising live microorganisms and selectively utilized substrates that benefit the host [167,168,169]. In the post-weaning intestine, probiotics may compete for nutrients and epithelial adhesion sites, acidify local habitats, produce bacteriocins, and contribute metabolites to microbial cross-feeding. Prebiotics favor microorganisms capable of utilizing the supplied substrates and modify the quantity and distribution of fermentation products. Depending on the formulation, the microbial and substrate components of a synbiotic may interact directly or through members of the resident microbiota. Gassericin A illustrates that bacteriocins can act beyond direct microbial antagonism: this microbiota-derived product acted on the porcine intestinal epithelium and increased resistance to early-weaning diarrhea [170]. Post-weaning galactooligosaccharide supplementation similarly altered hindgut microbial composition and fermentation, accompanied by improvements in intestinal morphology and growth [171].

Piglet studies further associate probiotic-induced ecological changes with mucosal regulation. In *E. coli*-challenged weaned piglets, a Ningxiang pig-derived *Enterococcus hirae* isolate reduced intestinal *E. coli* colonization, partially restored microbial community structure, increased acetate and propionate concentrations, improved barrier-associated indices, and reduced mucosal pro-inflammatory cytokine expression [172]. A Ningxiang pig-derived *Limosilactobacillus reuteri* increased colonic sIgA concentration, jejunal and ileal ZO-1 expression, and the ileal villus-height-to-crypt-depth ratio, while reducing IL-6 and TNF-α expression in the jejunum and colon [173]. Dietary *Lactiplantibacillus plantarum* increased ileal goblet-cell numbers and the mRNA expression of MUC1, occludin, and claudins 2–4, together with changes in jejunal and fecal microbial communities [174]. These effects may occur without permanent engraftment because transient persistence can be sufficient to modify microbial interactions, metabolite availability, and mucosal signaling.

Synbiotic combinations have also been evaluated in weaned piglets. In an ETEC K88 challenge model, raw potato starch combined with the probiotic *Escherichia coli* strains UM-2 and UM-7 improved growth, reduced diarrhea, and increased gut microbial diversity [175]. In another ETEC K88 challenge study, lactulose combined with *Lactiplantibacillus plantarum* increased the proportion of colonic butyrate, reduced plasma TNF-α and pig major acute-phase protein concentrations, and improved ileal villus height and goblet cell numbers [176].

### 7.3. Postbiotics and Microbial Metabolites

Postbiotics are preparations of inanimate microorganisms and/or their components that confer a health benefit on the host [177]. Such preparations contain inactivated microbial cells or cellular components and may also retain cell-wall structures, surface proteins, enzymes, peptides, and fermentation products. Purified microbial metabolites are considered separately, although both approaches deliver microbial signals without requiring the administered microorganism to remain viable or colonize the intestine. In F18-positive ETEC-challenged nursery pigs, a *Lactobacillus fermentate* attenuated mucosal inflammatory responses, supported epithelial integrity, and altered the mucosa-associated microbiota [178]. Dietary supplementation with postbiotics derived from *Pichia kudriavzevii* FZ12 reduced diarrhea, improved growth and intestinal indices, and increased intestinal *Lactobacillus* abundance in weaned piglets. Cytochrome c was subsequently identified as one active component that promoted the growth and biofilm formation of *Limosilactobacillus reuteri* FP13 in vitro [179]. A separate *Limosilactobacillus reuteri* postbiotic preparation increased fecal propionate and butyrate concentrations, improved serum antioxidant indices, and reduced mortality without affecting growth performance [180]. These findings indicate that cellular components and residual fermentation products may jointly contribute to the activity of complex postbiotic preparations.

Defined metabolites allow their epithelial and immune effects to be evaluated more directly. Dietary sodium butyrate reduced post-weaning diarrhea and intestinal permeability while altering bacterial communities [181]. Dietary lactate promoted epithelial proliferation and alleviated DSS-induced barrier injury through Wnt/β-catenin-associated signaling in weaned piglets, with complementary evidence obtained in IPEC-J2 cells [182]. Dietary indole-3-aldehyde promoted intestinal stem-cell expansion and improved mucus and tight-junction barriers, with similar proliferative effects observed in porcine intestinal organoids [58]. Indole-3-propionic acid showed a dose-dependent response: dietary supplementation at 50 and 100 mg/kg diet reduced diarrhea incidence and serum IL-6 and improved barrier-associated indices, whereas 600 mg/kg diet produced opposing inflammatory effects [183]. In addition, AhR-active metabolites contained in *Lacticaseibacillus rhamnosus* GG-derived extracellular vesicles activated porcine ILC3s and promoted IL-22 secretion in vitro, while oral *Lacticaseibacillus rhamnosus* GG administration alleviated PEDV-associated intestinal injury in piglets [79].

### 7.4. Functional Nutrients and Bioactive Compounds

Functional amino acids influence microbiota–mucosal immune interactions both as host nutrients and as precursors for microbial metabolism. Glutamine provides oxidative fuel for enterocytes and activated immune cells; in LPS-challenged piglets, supplementation altered the ileal microbiota and Th17/Treg-associated signaling while attenuating intestinal injury [184]. Tryptophan can enter host kynurenine and serotonin pathways or be converted by intestinal microorganisms into AhR- and PXR-active indole derivatives. Dietary tryptophan increased tight-junction proteins, sIgA, and porcine β-defensins in association with the enrichment of tryptophan-metabolizing bacteria [185]. A 2026 multi-omics study also reported reduced diarrhea, improved intestinal morphology, and coordinated changes in microbial composition and mucosal cytokine profiles following tryptophan supplementation [186]. Threonine is extensively utilized by the intestine for mucin and immunoglobulin synthesis [187], whereas arginine supports nitric oxide and polyamine production, epithelial repair, and cellular and humoral immunity [188]. By modifying mucus production, epithelial turnover, and immune-cell metabolism, these amino acids may also alter the nutritional and physicochemical environment encountered by the microbiota.

Plant- and fungal-derived bioactive compounds can act directly on host tissues or indirectly through changes in microbial composition and metabolism [189,190]. Polyphenols are often incompletely absorbed in the small intestine and undergo extensive microbial transformation in the hindgut, generating phenolic metabolites with distinct biological activities [191,192]. In deoxynivalenol-challenged weaned piglets, dietary resveratrol increased colonic *Roseburia* abundance and butyrate concentrations, reduced *Bacteroides* and unidentified Enterobacteriaceae, increased jejunal ZO-1 expression, and decreased TNF-α, IL-1β, and oxidative injury [193]. In ETEC K88-challenged weaned piglets, tannic acid improved barrier and immune indices, altered the colonic microbiota, and suppressed excessive TLR4/MyD88/NF-κB signaling [194]. Fungal-derived compounds may exert related effects through different chemical and biological mechanisms. In LPS-challenged piglets, *Cordyceps militaris* extract and cordycepin attenuated colonic injury and inflammatory responses in association with changes in microbial communities and pathways related to butyrate and amino acid metabolism [195].

Selected trace elements and bioactive peptides also modulate microbiota–mucosal immune interactions by influencing microbial metabolism and mucosal defense. Replacement of sodium selenite with biogenic selenium nanoparticles increased acetate and propionate concentrations and supported MUC2, tight-junction, antioxidant, and immune indices in early-weaned piglets [196]. Complementary gut-on-a-chip experiments, supported by mouse and early-weaned piglet models, further showed that biogenic selenium nanoparticles reinforced selenoenzyme defenses, maintained mitochondrial homeostasis, and suppressed mast-cell–epithelial inflammatory crosstalk and NLRP3-associated pyroptosis, thereby protecting the intestinal barrier against oxidative injury [197]. Antimicrobial peptides may influence microbiota–mucosal immune interactions by selectively suppressing susceptible bacteria and modulating epithelial barrier and immune responses. Dietary porcine intestinal antimicrobial peptide increased jejunal digestive-enzyme activities, intestinal alkaline phosphatase, and sIgA; reduced circulating D-lactate, diamine oxidase, and endotoxin; and increased the fecal abundance of *Limosilactobacillus reuteri* in weaned piglets [198]. In nursery Hainan piglets, dietary cecropin increased serum IgA and IgM, enhanced intestinal ZO-1 and occludin expression, and increased colonic *Lactobacillus* and *Limosilactobacillus* while reducing *Streptococcus* and *Escherichia–Shigella*, accompanied by a lower incidence of diarrhea [199].

## 8. Conclusions and Perspectives

Intestinal homeostasis in weaned piglets is maintained through reciprocal interactions among the gut microbiota, microbial metabolites, the epithelial barrier, and mucosal immunity. Microbial ligands and metabolites regulate epithelial renewal, barrier integrity, innate immune activity, and lymphoid responses. Conversely, mucus, antimicrobial peptides, sIgA, and cytokine-induced changes in the intestinal habitat influence which microorganisms persist, where they localize, and how closely they interact with the mucosa. Mucosal immunity therefore provides both protection against enteric pathogens and ecological selection within the intestinal community. The dietary, environmental, and developmental changes associated with weaning can destabilize these interactions, allowing barrier injury, inflammation, impaired colonization resistance, and pathogen expansion to reinforce one another. These disturbances contribute to post-weaning diarrhea, inefficient nutrient utilization, and reduced growth. Nutritional interventions should therefore be evaluated according to their capacity to restore specific microbial, metabolic, barrier, or immune functions rather than simply alter microbial composition or suppress individual inflammatory markers.

Several features of the available literature limit direct comparison. Studies differ in weaning age, basal diet, intervention dose and duration, intestinal sampling site and time point, and the type and severity of experimental challenge. These differences can influence microbial composition, barrier function, and immune responses. In addition, several detailed signaling mechanisms are based primarily on mouse models or in vitro systems and remain to be confirmed in piglets. This review brings these studies together narratively, but differences in study design and outcome reporting preclude pooled comparisons of intervention effects. Some of the apparent disagreement in the literature may therefore arise from experimental context rather than opposing biological responses.

Against this background, establishing causality remains a major challenge. Associations between bacterial taxa and cytokines, immune-cell states, sIgA responses, diarrhea, or growth do not establish the direction of regulation because both may reflect changes in diet, feed intake, barrier integrity, or pathogen exposure. Microbiota transplantation can demonstrate that a phenotype is transferable with a microbial community, but it cannot independently identify the responsible microorganism or molecular mechanism. Mechanistic studies should therefore connect a defined microbial strain or gene with its metabolite or molecular signal, the relevant host receptor and cell population, and a measurable intestinal phenotype. This requires controlled manipulation of microorganisms, microbial genes, metabolites, and host signaling pathways, supported by temporal and intestinal segment-specific analyses. Validation in piglets remains essential because microbial metabolic capacity, receptor expression, and immune maturation vary with intestinal location, developmental stage, and physiological state.

The translation of these mechanisms into nutritional practice also requires attention to developmental timing. Before weaning, access to solid feed may initiate microbial adaptation to plant-derived substrates and support mucosal maturation while milk-derived substrates and maternal immune factors remain available. Early probiotic or prebiotic exposure may similarly influence microbial assembly and mucosal development, although its effects depend on strain, substrate, dose, and duration. During the immediate peri-weaning period, reduced feed intake and unstable microbial fermentation increase the importance of protein digestibility, amino acid balance, appropriately selected fermentable carbohydrates, and barrier-supporting metabolites. As feed intake and hindgut fermentation recover, more complex fibers and prebiotics may support microbial cross-feeding, functional maturation, and ecological resilience. Responses nevertheless depend on the resident microbiota because its metabolic capacity determines whether dietary substrates are converted predominantly into SCFAs, lactate, succinate, indole derivatives, or proteolytic products. Barrier integrity, immune status, previous diet, and developmental age further contribute to variation among individual piglets. Nutritional strategies should therefore be matched to the function that is deficient, the physiological and microbial state of the recipient, and the developmental stage at which intervention is most likely to be effective.

## Figures and Tables

**Table 1:** Overall framework and key messages of this review.

Section	Guiding Question	Key Message
1. Introduction	Why are microbiota–mucosal immune interactions important during weaning?	Weaning exposes the immature intestine to major dietary, microbial, and environmental changes. Reciprocal interactions between the gut microbiota and mucosal immunity determine intestinal adaptation, diarrhea susceptibility, and growth performance.
2. Review Methodology	How was the evidence identified and synthesized?	A structured literature search was conducted with priority given to evidence from piglets. Non-porcine studies were included when necessary to explain mechanisms not yet established in pigs, and the evidence was synthesized thematically.
3. Post-weaning intestinal remodeling	What changes in the piglet intestine after weaning?	Weaning simultaneously reshapes the gut microbiota, epithelial barrier, and mucosal immune system, creating a vulnerable transition toward a new intestinal homeostatic state.
4. Microbial regulation of mucosal immunity	How does the gut microbiota regulate mucosal immunity?	Microbial components and metabolites regulate epithelial barrier function and innate and adaptive immune responses in weaned piglets.
5. Mucosal immune regulation of the microbiota	How does mucosal immunity shape the gut microbiota?	Mucus, antimicrobial peptides, sIgA, and cytokines control microbial localization, persistence, and interactions with the intestinal mucosa.
6. Dysregulation of microbiota–immune interactions	What happens when this bidirectional regulation is disrupted?	Barrier damage, excessive inflammation, loss of colonization resistance, and pathogen expansion reinforce one another, contributing to post-weaning diarrhea and impaired growth.
7. Nutritional modulation	How can nutrition restore microbiota–immune homeostasis?	Dietary substrates, microbiota-targeted interventions, microbial metabolites, and functional nutrients may restore microbial function, barrier integrity, and immune balance.
8. Conclusions and perspectives	What are the main research priorities?	Future studies should establish causal mechanisms in piglets and develop stage-specific nutritional strategies targeting defined microbial and immune functions.

## Data Availability

No new data were created or analyzed in this study. Data sharing is not applicable to this article.

## Abbreviations

The following abbreviations are used in this manuscript:

sIgA	secretory immunoglobulin A
ETEC	Enterotoxigenic *Escherichia coli*
SCFA	Short-chain fatty acid
DC	Dendritic cell
AhR	Aryl hydrocarbon receptor
IAld	Indole-3-aldehyde
ILC3	Group 3 innate lymphoid cell
LPS	Lipopolysaccharide
MAMP	Microbe-associated molecular pattern
MAPK	Mitogen-activated protein kinase
MyD88	Myeloid differentiation primary response 88
NF-κB	Nuclear factor kappa B
NLRP3	NLR family pyrin domain containing 3
NOD	Nucleotide-binding oligomerization domain
PEDV	Porcine epidemic diarrhea virus
pIgR	Polymeric immunoglobulin receptor
PPAR-γ	Peroxisome proliferator-activated receptor gamma
PXR	Pregnane X receptor
STAT3	Signal transducer and activator of transcription 3
Th17	T helper 17 cell
TLR	Toll-like receptor
Treg	Regulatory T cell
